# Do tumor cells escape from natural killer cell cytotoxicity by mimicking dendritic cells?

**DOI:** 10.18632/oncotarget.26815

**Published:** 2019-03-29

**Authors:** Hannah Wurzer, Antoun Al Absi, Céline Hoffmann, Clément Thomas

**Affiliations:** Clément Thomas: Department of Oncology, Cytoskeleon and Cancer Progression, Laboratory of Experimental Cancer Research, Luxembourg Institute of Health, Strassen, Luxembourg

**Keywords:** actin cytoskeleton, breast cancer, cytotoxicity, immune evasion, natural killer cells

Unraveling the molecular mechanisms underlying cancer immune evasion is an important step toward the development of more effective immunotherapies. Natural killer (NK) cells are cytotoxic effector lymphocytes of the innate arm of the immune system with potent activity in eliminating cancer cells. They can directly interact with cancer cells through a specific cell-cell interface termed the immunologic synapse. Activation of NK cell effector function is triggered by an excess of activating over inhibitory signals originating from receptor-ligand interactions between the two conjugated cells. Upon activation, NK cells engage in a series of well-defined cytoskeleton-dependent subcellular events leading to the directed secretion of lytic granules containing effector molecules, such as granzyme B and perforin, toward target cells [1]. In addition to their contact-dependent cytotoxicity, NK cells contribute to shape the adaptive antitumor immune response through production of chemokines and cytokines and activation of dendritic cells (DCs). Unlike CD8+ cytotoxic T lymphocytes (CTLs), NK cells do not require prior antigen exposure to recognize and kill transformed cells, and are accordingly considered as the first line of defense against cancer. However, to reach their full effector potential, NK cells require close interaction with dendritic cells (DCs). For instance, IL-15 production and trans-presentation by DCs was demonstrated to critically mediate NK cell activation *in vivo* [2]. In turn, NK cells promote DC maturation and functions by releasing pro-inflammatory cytokines in response to engagement of the NKp30 triggering receptor [3]. During their interaction with NK cells, mature DCs are protected from being killed by establishing a so-called “regulatory” synapse [4, 5]. A hallmark of the regulatory synapse is the prominent accumulation of filamentous actin in conjugated DCs near the cell-cell interface. Such accumulation was shown to facilitate inhibitory molecules polarization and/or stabilization at the synapse, and mediate widening of the synaptic cleft [4]. Preventing F-actin polymerization in DCs increased IFN-γ secretion and cytotoxicity by NK cells, and accordingly switched synapses from a regulatory to a cytolytic status.

**Figure 1:**
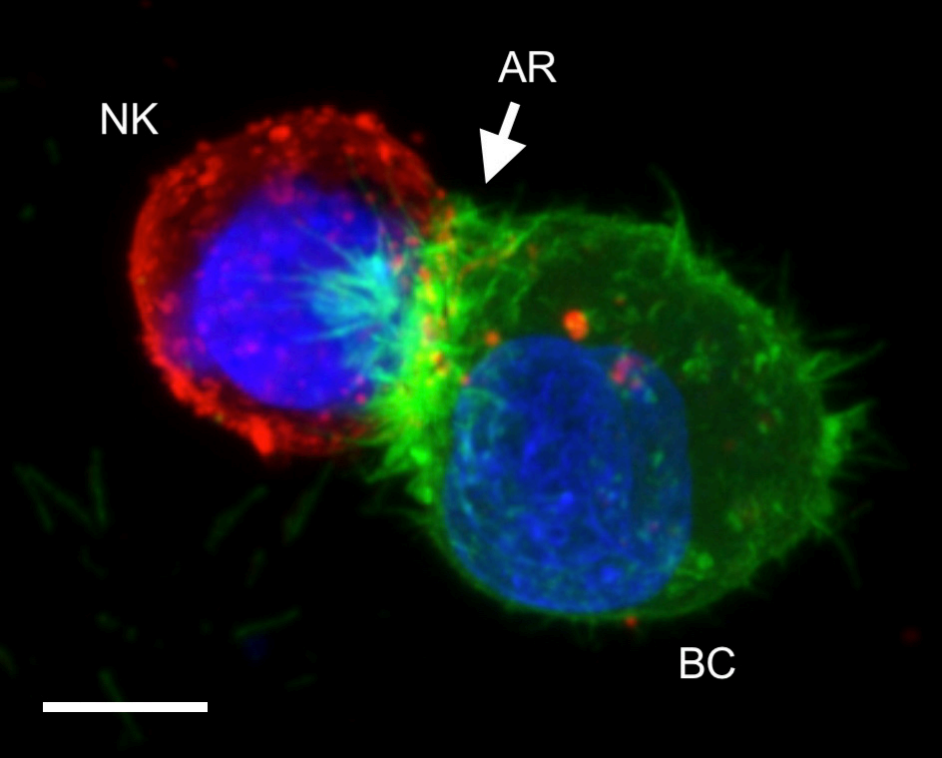
Confocal microscopy image showing a typical actin response (AR) in a LifeAct-EGFP expressing MDA-MB-231 breast cancer cell (BC) attacked by a natural killer cell (NK; PKH26 staining) Scale bar: 20 µm.

Recently, we reported on the pivotal role of the actin cytoskeleton in driving cancer cell resistance to NK cells [6]. Using a fluorescent actin reporter (LifeAct-EGFP [7]) that was introduced in various breast cancer cell lines, we found that a significant subpopulation of cells responds to NK-cell attack *via* rapid and massive accumulation of filamentous actin at the immunologic synapse, a process which was termed “actin response” (AR; Figure 1). Real time live-cell imaging provided direct evidence that cancer cells exhibiting an AR survive NK-cell attack, while tumor cells without an AR are efficiently killed. Moreover, inhibition of the AR by ablation of key regulators of the ARP2/3 complex, such as Cdc42 and N-WASP, was sufficient to convert resistant breast cancer cell lines to a highly susceptible phenotype. From a mechanistic standpoint, the AR leads to a remarkable reduction of NK cell-derived granzyme B in target cells. In addition, the AR is associated with a significant increase in density of key inhibitory ligands, including HLA-A, -B, -C and PD-L1, at the synaptic region of cancer cells. The causal relationship between actin-driven inhibitory ligand accumulation at the synapse and reduced levels of granzyme B in target cells warrants future investigation. However, it is tempting to propose that some cancer cells can trick NK cells by hijacking the protective mechanism normally used by mature DCs interacting with NK cells.

Animal experiments have been undertaken to validate the relevance of the AR to tumor immune evasion *in vivo*, and the molecular pathways underlying AR formation and downstream protective effects are under characterization. Our unpublished results support that actin remodeling in tumor cells is a fundamental process at the heart of several immune escape mechanisms, and represents a promising therapeutic point of intervention.
